# Clinical Characteristics and Risk of Diabetic Complications in Data-Driven Clusters Among Type 2 Diabetes

**DOI:** 10.3389/fendo.2021.617628

**Published:** 2021-06-30

**Authors:** Lin Xing, Fangyu Peng, Qian Liang, Xiaoshuang Dai, Junli Ren, Han Wu, Shufen Yang, Yaxin Zhu, Lijing Jia, Shancen Zhao

**Affiliations:** ^1^ BGI Institute of Applied Agriculture, BGI-Shenzhen, Shenzhen, China; ^2^ Endocrinology Department, Shenzhen People’s Hospital, Shenzhen, China

**Keywords:** diabetic complication, clinical characteristics, k-means, medication treatment, type 2 diabetes

## Abstract

**Background:**

This study aimed to cluster newly diagnosed patients and patients with long-term diabetes and to explore the clinical characteristics, risk of diabetes complications, and medication treatment related to each cluster.

**Research Design and Methods:**

K-means clustering analysis was performed on 1,060 Chinese patients with type 2 diabetes based on five variables (HbA1c, age at diagnosis, BMI, HOMA2-IR, and HOMA2-B). The clinical features, risk of diabetic complications, and the utilization of elven types of medications agents related to each cluster were evaluated with the chi-square test and the Tukey–Kramer method.

**Results:**

Four replicable clusters were identified, severe insulin-resistant diabetes (SIRD), severe insulin-deficient diabetes (SIDD), mild obesity-related diabetes (MOD), and mild age-related diabetes (MARD). In terms of clinical characteristics, there were significant differences in blood pressure, renal function, and lipids among clusters. Furthermore, individuals in SIRD had the highest prevalence of stages 2 and 3 chronic kidney disease (CKD) (57%) and diabetic peripheral neuropathy (DPN) (67%), while individuals in SIDD had the highest risk of diabetic retinopathy (32%), albuminuria (31%) and lower extremity arterial disease (LEAD) (13%). Additionally, the difference in medication treatment of clusters were observed in metformin (p = 0.012), α-glucosidase inhibitor (AGI) (p = 0.006), dipeptidyl peptidase 4 inhibitor (DPP-4) (p = 0.017), glucagon-like peptide-1 (GLP-1) (p <0.001), insulin (p <0.001), and statins (p = 0.006).

**Conclusions:**

The newly diagnosed patients and patients with long-term diabetes can be consistently clustered into featured clusters. Each cluster had significantly different patient characteristics, risk of diabetic complications, and medication treatment.

## Introduction

Diabetes is a chronic disease, not only has caused heavy social and economic burdens, but also prone to leading to multiple complications, which have profound impacts on the life quality of patients and may potentially cause death in severe cases. The prevalence of diabetes is rapidly increasing worldwide, so effectively preventing and managing diabetes has become an important topic at this stage (1, 2).

Diabetes is characterized by hyperglycemia, the causes of which are highly heterogeneous (3). Based on current classification criteria, diabetes is currently divided into two major subtypes, type 1 diabetes (T1D) and type 2 diabetes (T2D) which is approximately 85% (2, 4, 5). This classification relies on the age of disease diagnosis; however, it may not be enough to characterize complications and outcomes for subtypes. Individuals with diabetes have a different natural course of hyperglycemia, therefore theoretically should be treated with different clinical strategies better fitted their metabolic characteristics (6, 7). A novel approach to detailed characterize the diabetes population and explore the clinical features can be very beneficial to aid with the treatment of diabetes patients.

In recent years, novel stratifications of diabetes have been attempted worldwide. Three subgroups of T2D were identified using a topological analysis based on patient–patient networks (8). It is a valuable attempt to classify the patients, however, because the approach required genotype data from patients, this can be difficult to implement in clinical settings. Moreover, in Ahlqvist and colleagues’ study, five replicable clusters of diabetes based on six common clinical variables were found, which included glutamic acid decarboxylase autoantibody (GADA), HbA1c, BMI, age at onset of diabetes, and homeostasis model estimates of β-cell function (HOMA2B) and insulin resistance (HOMA2IR) (9). The five diabetes clusters were cluster 1, severe autoimmune diabetes (SAID); cluster 2, severe insulin-deficient diabetes (SIDD); cluster 3, severe insulin-resistant diabetes (SIRD); cluster 4, mild obesity-related diabetes (MOD); and cluster 5, mild age-related diabetes (MARD). Cluster 1 was characterized by the presence of GADA, being similar to T1D, and the other four clusters were T2D with the absence of GADA positivity and the other five variables. Safai et al. (10) used similar routine clinical markers to sub-group the patients into five clusters and reported the difference in probability of diabetes complications, such as cardiovascular disease, nephropathy and neuropathy. In Zaharia et al. (11), patients in German with newly diagnosed type 1 or type 2 diabetes were grouped into the same five clusters, validating such techniques in different population. In addition, the five-year follow-up research also reported the different prevalence in subgroups in terms of non-alcoholic fatty liver disease and diabetic neuropathy. In Dennis et al. (12), the five clusters could be replicated and the difference in glycemic progression were identified. Ahlqvist and colleagues in 2020 revisited the sub-grouping technique in several populations and reported the difference among groups for diabetic complications, such as retinopathy, neuropathy, kidney disease and fatty liver (13). To alleviate the requirement on clinical markers, in Kahkoska et al. (14), three global trails (DEVOTE, LEADER, and SUSTAIN-6) with recent-onset diabetes were tested for the clustering technique based on three variables, age at diabetes diagnosis, baseline glycated hemoglobin (HbA1c) and body mass index (BMI). The four T2D clusters could be fully replicated and the risk of major adverse cardiovascular events and death differed significantly in the follow-up duration. In addition, the risk of nephropathy differed in clusters. With clinical variables, HbA1c, BMI, age at onset of diabetes, HOMA2B and HOMA2IR, there were attempts that clustering the newly diagnosed diabetes patients in the United States and China into four subgroups and confirmed that different ethnic groups can also be clustered by the same variables (15). For many previous researches that had already proven the promising application of such sub-grouping techniques in precision medicine, the clustering target were usually newly diagnosed patients. Since the applications of sub-grouping technique on different populations were proven to be quite robust, we would like to make some exploration of the clustering techniques on both newly diagnosed and long-term diabetes patients, aiming to facilitating the application domain as many admitted patients were in various stages.

Current diabetes treatments mainly focus on controlling blood glucose levels, but with many researches elucidated specific characteristics with subgroups of T2D, more informative treatments on diabetes related issues, such as kidney, cardiovascular and cerebrovascular diseases, became promising. We believe a precise characterization of T2D patient populations in the clinical settings would be beneficial for the understanding of T2D pathophysiology and improvement of clinical management. Therefore, the objectives of this study are to cluster newly diagnosed and previously known Chinese T2D patients, and to explore the clinical characteristics, the risks of diabetic complications, and medication treatment in each cluster. We aim to identify different subgroups of T2D patients through the K-means clustering method with five commonly used clinical variables including HbA1c, BMI, age at diagnosis, HOMA2-B, and HOMA2-IR, and then compare clinical characteristics, identify individuals with increased risk of complications, and distinguish different medication treatment in each subgroup.

## Materials and Methods

### Study Population

This was a cross-sectional study conducted from January 2018 to November 2019 at the No.1 Shenzhen People’s Hospital. Medical records of 1,240 participants were collected on a first come first chosen basis within a one-year time window, which include anthropometric measurements, laboratory tests, complication diagnostic information, and medication regime. All participants, diagnosed with type 2 diabetes, involved in this study were aged 18 years and above. This study was approved by the medical research ethics committee at Shenzhen People’s Hospital. Informed consent was obtained from the participants subjected to anonymous information utilization in medical research.

### Measurements

The height and weights of participants were measured using an automatic anthropometer and the body mass index (BMI) was calculated as body weight/height (kg/m^2^). Blood pressure was measured by trained nurses with a blood pressure monitor. Laboratory measurements were taken in a fasting state following the standardized procedures during the health examination. Biochemical indices, such as fasting blood glucose (FPG), urine acid (UA), total cholesterol (TC), triglycerides (TG), high-density lipoprotein (HDL) cholesterol and low-density lipoprotein (LDL) cholesterol, were measured by the hexokinase method and C-peptide (CP) concentrations were measured by radioimmunoassay. The FPG and CP were used to calculate homeostasis model assessment 2 estimates of insulin resistance (HOMA2IR) and homeostasis model assessment 2 estimates of β-cell function (HOMA2B) with the HOMA2 calculator v2.2.3 at www.dtu.ox.ac.uk (16).

### Definitions of Diabetes and Diabetic Complications

The criteria to diagnose participants to have diabetes followed the internationally adopted standards set by the World Health Organization (WHO) Diabetes Expert Committee in 1999 (17). Estimated glomerular filtration rate (eGFR) was calculated using the chronic kidney disease epidemiology collaboration (CKD-EPI) equation, which was used to classify kidney function as normal (stage 1, eGFR >90 ml/min per 1.73 m^2^), abnormal (stage 2, eGFR 60–90 ml/min per 1.73 m^2^ or stage 3, eGFR <60 ml/min per 1.73 m^2^) (18, 19). The range of urinary albumin to creatinine ratio (UACR) was used to describe the Albuminuria progression. The UACR less than 30, between 30 and 300, and above 300, were defined as normal, microalbuminuria, and macroalbuminuria, respectively. Ankle-brachial index (ABI) measurements were taken after a five-minute break with the supine position, which were used to identify the lower extremity arterial disease (LEAD) including hardened vessels and arterial occlusion (20). The ABI values between 0.9 and 1.3, larger than 1.3 or less than 0.9 were considered as normal and abnormal arterial, respectively (21). Both diabetic retinopathy and diabetic peripheral neuropathy (DPN) were defined following the American Diabetes Association’s criteria (22, 23). The diagnosis of DPN was based on the multiple symptoms and diabetes history. For the impact on the small fibers, the symptoms usually involved pain and dysesthesia, which can be assessed with the pinprick and temperature sensation tests. As the impact developed on large fibers, the symptoms usually involved numbness and loss of protective sensation, which can be confirmed by the vibration perception and 10-g monofilament tests. For diabetic retinopathy, the diagnosis was based on an initial dilated and comprehensive eye examination performed by an ophthalmologist or optometrist. Patients were arranged for diagnosis examinations during the first visit, based on which the initial status of diabetic retinopathy was determined (22, 23).

### Cluster Analysis

**Figure 1:**
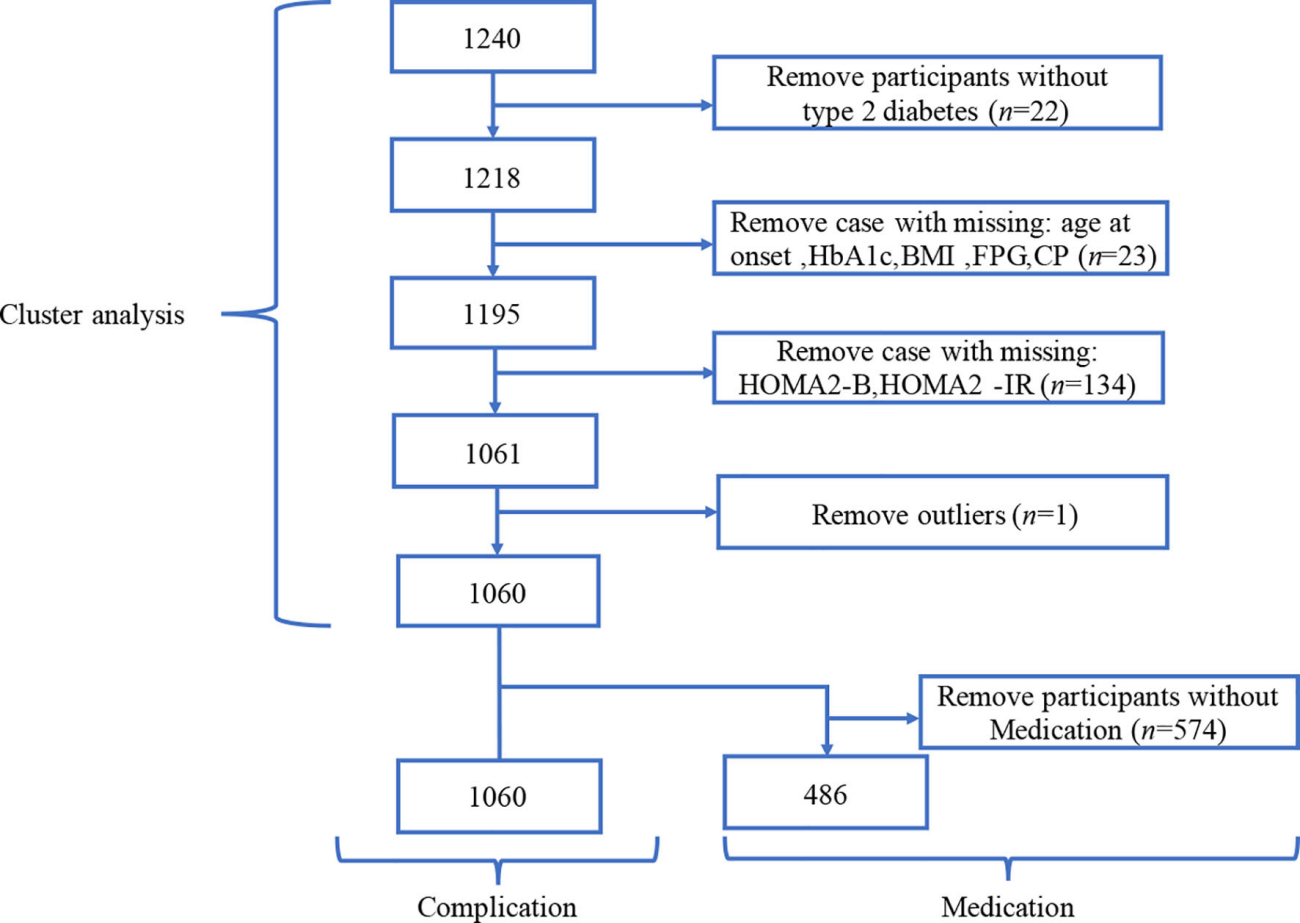
Flowchart for the selection of participants in the different analysis section.

The data cleaning process followed four steps. Step-1, twenty-two participants without type 2 diabetes were removed from the dataset as they were not part of the target group. Step-2, twenty-three individuals with missing information in variables, such as BMI, HbA1c, and so on, were removed. Step-3, To improve the group clustering quality, one hundred thirty-four participants with values beyond the defined range of HOMA2 calculators were removed before the feature engineering. Step-4, by checking the variables individually, one extreme outlier was identified and removed. After the data cleaning procedures, 1,060 participants were included in cluster analysis, with the result of which two additional analyses focused on medication usage and diabetes complications were carried out. For the comparison of diabetes complications, 1,060 participants were included in the analysis. For the medication usage comparison, after the removal of participants that did not have the required information, 486 participants were included in medication analysis (**Figure 1**).

### Statistical Analysis

The k-means was used to cluster the data according to five variables, age at diagnosis, BMI, HbA1c, HOMA2B, and HOMA2IR. All data were scaled to mean zero and unit variance before clustering. K-means clustering was performed using the Hartigan and Wong algorithm implemented in the ‘k-means’ package and the optimal number of clusters was determined by elbow method from ‘NbClust’ package in R. To use the elbow method in the given data set, the number of clusters were plotted against the total explained variance. With a straight line going across the different number of clusters, the point where the increase of variance explained became slow was the target. The number of clusters corresponding to the point was the reasonable number of clusters. Based on the characteristics of clusters described by Ahlqvist et al. (9), patients were assigned to explainable clusters, severe insulin-deficient diabetes (SIDD), severe insulin-resistant diabetes (SIRD), mild obesity-related diabetes (MOD), or mild age-related diabetes (MARD). For descriptive statistics between subgroups, the chi-square test was used for categorical data. Skewed data were log-transformed before analysis. To account for the impact of age, the linear model incorporated age variable as correction factor when comparing the clinical features in subgroups. To understand the impact of gender on the diabetes-related complication of subgroups, odds ratios (ORs) were modeled using logistic regression. P-values less than 0.05 were considered statistically significant. Statistical analyses were done with R version 3.5.3.

## Results

### Cluster Analysis

General characteristics of the population and clinical data for these patients are shown in **Table 1**. In this study, the age of participants ranged from 24 to 99, with most individuals being male (61%). According to the onset of the patient’s diabetes, around 16% of the patients were newly diagnosed diabetes, and 84% of the patients were long-term diabetes (**Figure S1**).

**Table 1:** Characteristics of the participants in allocated clusters.

	All	SIDD	SIRD	MOD	MARD
N	1,060	223	225	260	352
Male, N (%)	644 (61)	126 (57)	150 (66)	184 (70)	188 (53)
Female, N (%)	416 (39)	97 (43)	76 (34)	76 (30)	164 (47)
Age (years)	57.0 (50.0–65.0)	59.3 (53.0–68.0)	60.0 (52.0–66.1)	50.0 (43.8–57.2)	58.6 (53.0–66.0)
Diabetes duration (years)	9.9 (3.9–15.0)	7.9 (2.0–11.0)	8.0 (3.0–13.0)	10.0 (5.0–17.0)	10.0 (3.9–16.0)
Age at diagnosis (years)	47.0 (39.7–54.0)	51.9 (44.0–59.0)	50.0 (43.0–58.0)	38.2 (33.0–43.0)	48.1 (42.3–54.1)
Fasting blood glucose (mmol/L)	7.2 (5.7–9.4)	10.2 (7.7–13.1)	5.6 (5.0–6.4)	7.0 (8.5–10.5)	5.9 (6.8–7.9)
C-peptide (nmol/L)	1.8 (1.3–2.4)	1.4 (0.9–1.9)	2.6 (2.1–3.0)	1.4 (1.9–2.4)	1.8 (1.6–2.0)
SBP (mmHg)	126 (115–140)	129 (116–148)	125 (115-139)	126 (115–138)	125 (113–138)
DBP (mmHg)	77 (70–85)	80 (73–88)	77 (70-84)	80 (73–87)	75 (68–82)
eGFR (ml/min/1.73m^2^)	93.9 (78.6–104.1)	95.8 (80.2–106.1)	86.1 (70.0-98.0)	99.7 (86.2–110.4)	93.6 (79.3–100.9)
Urine acid (μmol/L)	338.0 (281.0–401.3)	305.5 (252.0–357.3)	369.0 (310.5-425.8)	358.0 (308.5-415.8)	326.0 (266.8–395.0)
UACR (mg/g)	7.3 (3.2–22.9)	11.1 (5.5–42.7)	6.2 (2.7–15.0)	8.2 (3.8–28.7)	5.5 (1.8–17.3)
Total cholesterol (mmol/L)	4.6 (3.9–5.5)	5.0 (4.2–5.9)	4.1 (3.5–4.9)	4.7 (3.8–5.5)	4.7 (4.0–5.5)
Triglycerides (mmol/L)	1.4 (1.0–2.1)	1.5 (1.0–2.2)	1.4 (1.0–1.9)	1.7 (1.2–2.8)	1.2 (0.9–1.7)
HDL-cholesterol (mmol/L)	1.1 (0.9–1.4)	1.1 (0.9–1.3)	1.1 (0.9–1.3)	1.0 (0.9–1.2)	1.3 (1.0–1.5)
LDL-cholesterol (mmol/L)	2.7 (2.1–3.4)	3.1 (2.4–3.8)	2.4 (1.9–3.1)	2.8 (2.1–3.5)	2.7 (2.1–3.4)

All values were N (%) or median (IQR). SIDD, severe insulin-deficient diabetes; SIRD, severe insulin-resistant diabetes; MOD, mild obesity-related diabetes; MARD, mild age-related diabetes; HOMA2B, homeostatic model assessment 2 estimates of β-cell function; HOMA2IR, homeostatic model assessment 2 estimates of insulin resistance; SBP, systolic blood pressure; DBP, diastolic blood pressure; eGFR, estimated glomerular filtration rate; UACR, urinary albumin to creatinine ratio.

**Figure 2:**
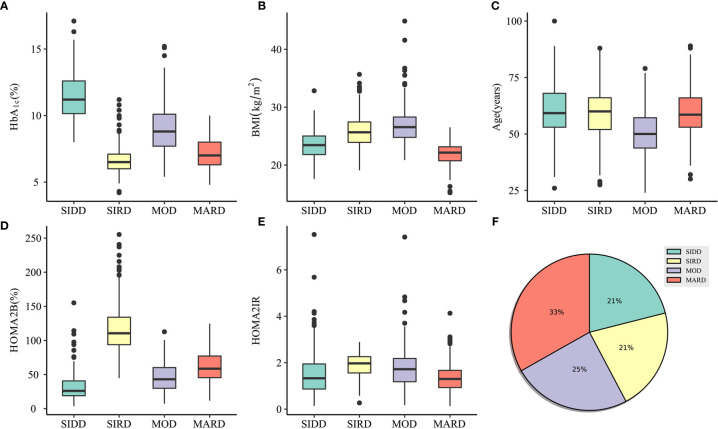
Characteristics of clusters and distribution of participants. **(A–E)** The distribution of HbA1c, BMI, Age, HOMA2B, and HOMA2IR in SIDD, SIRD, MOD, and MARD clusters. **(F)** Distribution of patients (*n* = 1060) in subgroups clustered by k-means approach. SIDD, severe insulin-deficient diabetes; SIRD, severe insulin-resistant diabetes; MOD, mild obesity-related diabetes; MARD, mild age-related diabetes; HOMA2B, homeostatic model assessment 2 estimates of β-cell function; HOMA2IR, homeostatic model assessment 2 estimates of insulin resistance.

One thousand sixty patients were classified into four clusters, each of which had distinctive clinical features (**Figure 2** and **S2**). Cluster 1, including 21% of the patients characterized by high age, low BMI, low insulin secretion (low HOMA2B index), and poor metabolic control (the highest HbA1c and Fasting blood glucose level), was identified as severe insulin-deficient diabetes (SIDD). Cluster 2 included 21% of patients who were labeled as severe insulin-resistant diabetes (SIRD), which was characterized by insulin resistance (high level of HOMA2-B and HOMA2-IR), high BMI, and good metabolic control. Cluster 3, including 25% of patients, was labeled as mild obesity-related diabetes (MOD) characterized by obesity, low age, average β-cell function, and insulin resistance. Cluster 4, the largest subgroup (33%), was labeled as mild age-related diabetes (MARD), which include older participants with modest metabolic control, insulin resistance, β-cell function, and the lowest BMI.

### Comparison of Clinical Features in Subgroups

The clinical characteristics of subgroups can be found in **Table 1**. In this section, the study mainly focused on the characteristics of subgroups in blood pressure, renal function, and lipids. Blood pressure, systolic blood pressure (SBP), and diastolic blood pressure (DBP) were following a similar trend, being highest in SIDD subgroup. Besides, in terms of DBP levels, there was no significant difference between SIRD and MARD (p = 0.104) (**Tables S1, S2**).

Regarding renal function, patients in SIRD had the lowest eGFR and highest urine acid (UA) compare to those in other subgroups. Urinary albumin to creatinine ratio (UACR) was highest in MOD cluster whereas there no statistical significance in MARD compared to SIDD (p = 0.174) and SIRD (p = 0.334), respectively (**Table S5**).

Concerning lipids, total cholesterol (TC) was the highest in patients assigned to the SIDD cluster and being statistically higher than MARD (p <0.001) whereas there was no significant difference between MOD and MARD (**Table S6**). Patients in MARD subgroup had the lowest triglycerides (TG) and highest HDL-cholesterol (HDL) levels comparable to those in other subgroups (**Tables S7–S8**). LDL-cholesterol (LDL) was the lowest in patients assigned to the SIRD cluster compared with all other clusters and there was no statistical significance between MOD and MARD clusters (p = 0.373) (**Table S9**).

### Comparison of Diabetes Complications in Subgroups

**Figure 3:**
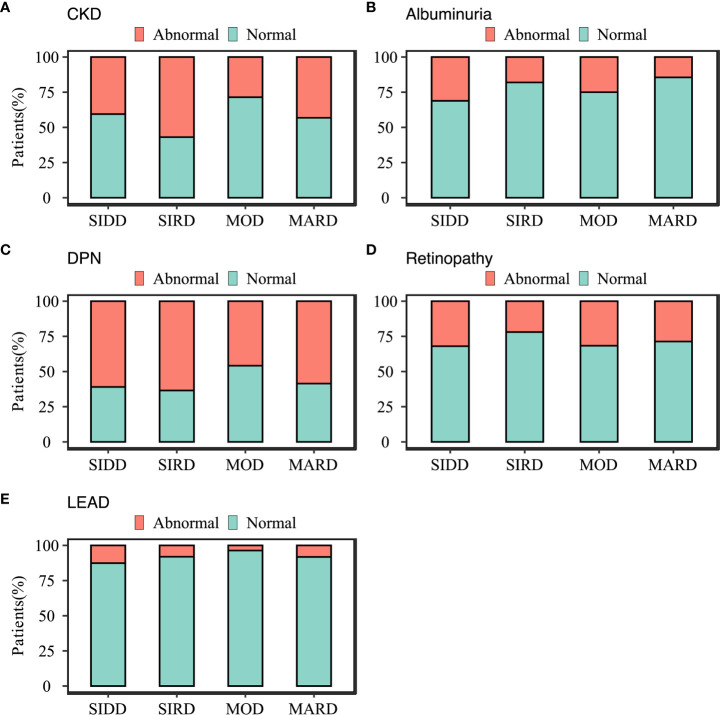
Characteristics of diabetes-related complications in subgroups. **(A)** chronic kidney disease; **(B)** albuminuria; **(C)** diabetic peripheral neuropathy; **(D)** diabetic retinopathy; **(E)** Lower-extremity arterial disease diabetes-related complications. SIDD, severe insulin-deficient diabetes; SIRD, severe insulin-resistant diabetes; MOD, mild obesity-related diabetes; MARD, mild age-related diabetes.

The differences in the prevalence of diabetes complications between the subgroups were shown in **Figure 3**. Patients in SIRD had the highest prevalence of stages 2 and 3 CKD in the SIRD cluster (57%) compared to those in SIDD (41%; p = 0.001), MOD (29%; p <0.001) and MARD (43%; p= 0.002) (**Figure 3A**). The SIDD cluster, being similar to MOD (p = 0.172), had the highest prevalence of albuminuria (31%), compared to those in SIRD (18%; p = 0.002) and MARD (14%; p <0.001) (**Figure 3B**). Patients in the MOD (48%) cluster had lowest prevalence of DPN (**Figure 3C**) compared to those in SIDD (60%; p = 0.02), SIRD (67%, p = 0.001) and MARD (63%; p = 0.003). The prevalence of retinopathy was highest in SIDD (32%) and MOD (32%) (**Figure 3D**), but there was no significant difference between the rest groups (p >0.05). The LEAD had the highest prevalence in SIDD (13%), but not being significantly different from SIRD (8%; p = 0.209) and MARD (8%; p = 0.168) (**Figure 3E**).

Separate analyses stratified by gender were performed to illustrate the risk trends of diabetes-related complications in subgroups. For male participants, there was no significantly different (p >0.05) in albuminuria and retinopathy among subgroups (**Table 2**). In other complications, the MARD consistently had a higher risk of disease onset compared to other subgroups (ORs >1; p <0.05). For female participants, the risk of diabetes complication, retinopathy, was similar among subgroups (p >0.05), whereas, in other subgroups, the risk trends varied (**Table 2**).

**Table 2:** Association between diabetes-related complications and clusters in different gender stratification.

Complication	Cluster	Male OR	P-value	Female OR	P-value
CKD	MOD	1.00	–	1.00	–
	MARD	2.28 (1.47–3.54)	<0.001	1.53 (0.85–2.83)	0.162
	SIDD	1.77 (1.09–2.90)	0.021	1.62 (0.84–3.16)	0.148
	SIRD	3.47 (2.18–5.58)	<0.001	3.02 (1.55–6.03)	0.001
Albuminuria	MARD	1.00	–	1.00	–
	MOD	1.55 (0.92–2.61)	0.096	2.83 (1.39–5.82)	0.004
	SIDD	1.69 (0.96–2.98)	0.066	4.70 (2.49–9.15)	<0.001
	SIRD	1.12 (0.62–1.99)	0.698	1.55 (0.70–3.35)	0.261
DPN	MOD	1.00	–	1.00	–
	MARD	1.89 (1.22–2.94)	0.004	1.34 (0.73–2.48)	0.339
	SIDD	1.40 (0.88–2.24)	0.154	2.70 (1.37–5.39)	0.004
	SIRD	1.78 (1.11–2.89)	0.017	2.57 (1.25–5.39)	0.011
Retinopathy	SIRD	1.00	–	1.00	–
	MARD	1.37 (0.72–2.63)	0.334	1.49 (0.69–3.33)	0.311
	MOD	1.60 (0.88–2.96)	0.125	1.79 (0.78–4.23)	0.171
	SIDD	1.43 (0.76–2.74)	0.271	2.05 (0.95–4.63)	0.071
LEAD	MOD	1.00	–	1.00	–
	MARD	3.34 (1.25–10.50)	0.023	1.23 (0.32–5.88)	0.768
	SIDD	3.00 (1.03–9.89)	0.050	4.14 (1.27–18.66)	0.032
	SIRD	2.43 (0.78–8.23)	0.128	1.93 (0.48–9.49)	0.368

Male OR, odds ratio in population with only male gender; Female OR, odds ratio in population with only female gender; SIDD, severe insulin-deficient diabetes; SIRD, severe insulin-resistant diabetes; MOD, mild obesity-related diabetes; MARD, mild age-related diabetes; CKD, Chronic kidney disease; DPN, Diabetic peripheral neuropathy; LEAD, Lower-extremity arterial disease. In each comparison, the reference group was identified with OR being 1.

### Comparison of Medication Application in Subgroups

The detailed comparison of nine types of glucose-lowering drugs, statins, and antihypertensive in different subgroups were shown in **Table 3**. Significant differences in treatment between the subgroups were observed in metformin (p = 0.012), α-glucosidase inhibitor (AGI) (p = 0.006), dipeptidyl peptidase 4 inhibitor (DPP-4) (p = 0.017), glucagon-like peptide-1 (GLP-1) (p <0.001), insulin (p <0.001), statins (p = 0.006).

**Table 3:** Characteristics of medication application by allocated clusters.

	All	SIDD	SIRD	MOD	MARD	*P value*
Medication, N (%)	486 (100)	75 (15)	106 (22)	109 (22)	196 (40)	–
Metformin, N (%)	362 (74)	54 (72)	78 (74)	94 (86)	136 (69)	0.012
SU, N (%)	60 (12)	9 (12)	15 (14)	12 (11)	24 (12)	0.917
TZDs, N (%)	15 (3)	1 (1)	5 (5)	6 (6)	3 (2)	0.139
AGI,N (%)	223 (46)	43 (57)	40 (38)	40 (37)	100 (51)	0.006
DPP-4,N (%)	270 (56)	37 (49)	53 (50)	54 (50)	126 (64)	0.017
SGLT-2,N (%)	84 (17)	10 (13)	18 (17)	21 (19)	35 (18)	0.760
GLP-1,N (%)	24 (5)	1 (1)	4 (4)	17 (16)	2 (1)	<0.001
Glinides, N (%)	21(4)	3 (4)	8 (8)	5 (5)	5 (3)	0.239
Insulin, N (%)	192 (40)	57 (76)	21 (20)	49 (50)	65 (33)	<0.001
Statins, N (%)	326 (67)	61 (81)	62 (59)	78 (72)	125 (64)	0.006
AHT, N (%)	195 (40)	34 (45)	51 (48)	39 (36)	71 (36)	0.124

All values were N (%). SU, sulphonylurea; TZDs, thiazolidinediones; AGI, α-glucosidase inhibitor; DPP4-I, dipeptidyl peptidase 4 inhibitors; SGLT2, sodium-glucose co-transporter 2 inhibitors; GLP-1, glucagon-like peptide-1; AHT, antihypertensive treatment (includes diuretics, β-blockers, and renin–angiotensin system inhibitors); SIDD, severe insulin-deficient diabetes; SIRD, severe insulin-resistant diabetes; MOD, mild obesity-related diabetes; MARD, mild age-related diabetes.

Patients in the SIDD cluster had the highest proportion of Insulin (76%), AGI (57%), and statins (81%) treatments compared to those in other subgroups. The MOD cluster had the highest proportion of metformin treatment (86%), compared with SIDD (72%), SIRD (74%), and MARD (69%). Patients in MOD cluster also had the highest percentage of GLP-1 treatment (16%), compared with those in SIDD (1%), SAID (4%), and MARD (1%). Moreover, DPP-4 treatment was most frequently used in MARD subgroup (64%).

## Discussion

In this study, the data-driven approach to distinguish the status of diabetes was reproducible and the distribution of patients was similar to that of the Swedish cohort (9). To improve the comprehensive application of such clustering approach in clinical settings, this study tested on population included both newly diagnosed patients and patients with long-term diabetes, which is different from previous studies that only covered newly diagnosed individuals (9, 11, 12, 15). Through the exploration on mixed population, we hope to extend the approach closer to clinical practices as many admitted patients were not newly diagnosed. To evaluate the performance of the clustering technique on data set, we checked the mathematical clustering stability and interpretation of the grouped clusters. Overall, the data driven approach could be used on the mixed population and yielded decent stability. Notably, the clinical characteristics of clusters in this study were similar to previous researches, with a slightly higher proportion of patients in the severe insulin-deficient diabetes (SIDD) cluster (21%) and severe insulin-resistant diabetes (SIRD) cluster (21%). This discrepancy may result from the higher proportion of hospitalized and severe participants in our study than other research population.

The highest prevalence of stages 2 and 3 chronic kidney disease (CKD) was in the SIRD subgroup with the lowest eGFR and highest urine acid. The possible explanation may be that the significant feature of the SIRD group is insulin resistance, which could lead to water-sodium retention, glomerular hypertension, hyperfiltration, and hyperuricemic, thus accelerating the progression of CKD. The trend of risk levels associated with CKD was different in male and female gender (**Table 2**). There were more subgroups associated with increased risk of CKD in males compared to females, which leads to a possible belief that the prognosis of CKD may depend on gender. This was confirmed by research showing that the female gender was associated with a slower decline in GFR and better patient and renal survival in a 10-year following-up study (24). In 2016, summarized multiple publication papers related to CKD research, Goldberg and Krause reported that the mortality risk of CKD in males was higher than in females (25).

Diabetic retinopathy, presented in approximately 30% of the individuals with diabetes (26), occurred most frequently in SIDD (32%) and mild obesity-related diabetes (MOD) (32%). SIDD, together with MOD, had the highest levels of fasting blood glucose and blood pressure (BP). Multiple studies showed that hyperglycemia and hypertension are risk factors for retinopathy and were different explanations regarding the connections. One possible reason is metabolic pathways triggered by hyperglycemia in diabetes, such as the polyol and the hexosamine pathways, the *de novo* synthesis of diacyl-glycerol, and advanced glycosylation end products (AGEs), can promote the development of retinopathy (27). Another possible reason is that hypertension increases the expression of pro-inflammatory molecules in the retina (28). The albuminuria, which is a powerful predictor of renal and cardiovascular risk (29), especially microalbuminuria, was highest in the SIDD subgroup. Since hypertension and hypercholesterolemia are causal risk factors of cardiovascular diseases as well (30), it is no surprise to have the SIDD group with a high rate of cardiovascular diseases. Based on the literature, retinopathy can often precede diabetic nephropathy in patients with T2D and this was confirmed by the clustering result that SIDD subgroup has the highest prevalence on both albuminuria and retinopathy. In terms of gender effect, there was a study indicating that the female showed a significantly higher prevalence and the female gender was an independent factor of disease development (31).

The prevalence of lower extremity arterial disease (LEAD) was highest in the SIDD subgroup (13%). Moreover, the retinopathy and albuminuria also had the highest prevalence in SIDD and there was literature indicating that they may be independent risk factors for LEAD (32–34). Similar to other researches, this study found the blood pressure and blood lipids were key risk factors for LEAD, being highest in SIDD (34, 35).

Regarding medication strategy, subgroups had different treatments, being consistent with the physiological characteristics of patients. The proportion of insulin, AGI, and statins was significantly higher in SIDD compared with other subgroups. On the other hand, individuals in SIDD hold very low β-cell reserves (the level of HOMA2-B was lowest), thus were treated preferentially with insulin. AGI drugs can reduce the amounts of insulin needed to control postprandial hyperglycemia by slowing down the digestion of complex carbohydrates and sucrose, therefore it is suitable for the insulin-deficient characteristic (36). The possible reason for the high prevalence application of statins in SIDD may result from the treatment of prevailed hyperlipidemia conditions.

In terms of MOD, having the highest BMI and mild diabetic symptoms, metformin, and GLP-1 were the medications used most frequently. Studies have shown that weight loss can effectively control diabetes’s disease course (37). As metformin and GLP-1 have significant effects on weight loss, the medications are suitable for MOD. DDP-4 is characterized as a low risk of hypoglycemia, a high compliance rate, therefore it is suitable for age-related diabetes (MARD) (38). The SIRD subgroup, characterized by the most severe insulin resistance and best β-cell function, was treated most frequently with metformin drugs. As metformin could increase insulin sensitivity, the application of insulin in this subgroup was lowest among all subgroups (39). Overall, the utilization of metformin, as the first-line treatment of type 2 diabetes, had the highest proportion in four subgroups, all above 69.4%.

To extend our study and comprehensively aid with clinical treatment on diabetes, long-term follow-up studies are necessary to explore disease progression and the treatment response. Additionally, newly discovered biomarkers with advanced techniques including genomics, transcriptomics, gut microbiota, could be considered to refine the current stratification strategy. To make the approach being feasible to apply in clinical conditions, a decision-making support system is necessary.

In conclusion, this study testified that the data-driven approach to cluster both newly diagnosed patients and patients with long-term diabetes can yield a consistent result. Each cluster had significantly different patient characteristics, risk of diabetic complications, and medication treatment. These findings have a potential value for clinical trial enrolment and early treatment stratification.

## Data Availability Statement

The raw data supporting the conclusions of this article will be made available by the authors, without undue reservation.

## Ethics Statement

The studies involving human participants were reviewed and approved by the Medical Research Ethics Committee at Shenzhen People’s Hospital. The patients/participants provided their written informed consent to participate in this study.

## Author Contributions

LX and FP performed data analysis, interpretation and manuscript writing.QL supervised the data collection and research collaboration. XD, JR, HW and SY participated in data collection and literature search. LJ and SZ designed the experiment and supervised the overall progress. All authors contributed to the article and approved the submitted version.

## Conflict of Interest

Authors LX, FP, XD, JR, and SZ were employed by company BGI-Shenzhen.

The remaining authors declare that the research was conducted in the absence of any commercial or financial relationships that could be construed as a potential conflict of interest.
