# Supplementary material for: Clinical Characteristics and Risk of Diabetic Complications in Data-Driven Clusters Among Type 2 Diabetes
Source: Front Endocrinol (Lausanne). 2021 Jun 30;12:617628. doi: 10.3389/fendo.2021.617628 (PMC8281969; doi:10.3389/fendo.2021.617628)
Supplement: Supplementary file 1 [file DataSheet_1.docx]

Supplementary Material

# Supplementary Figure

**
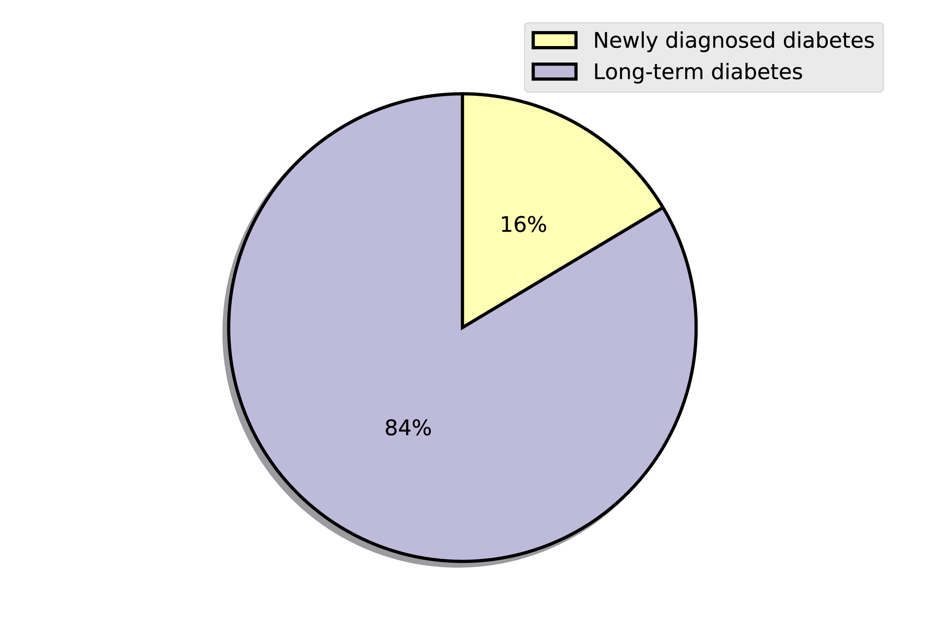
**

**Fig. S1.** Patient distribution according to the duration of diabetes. Newly diagnosed diabetes, the duration of patient’s diabetes is less than two years; Long-term diabetes, the duration of patient’s diabetes is more than two years.


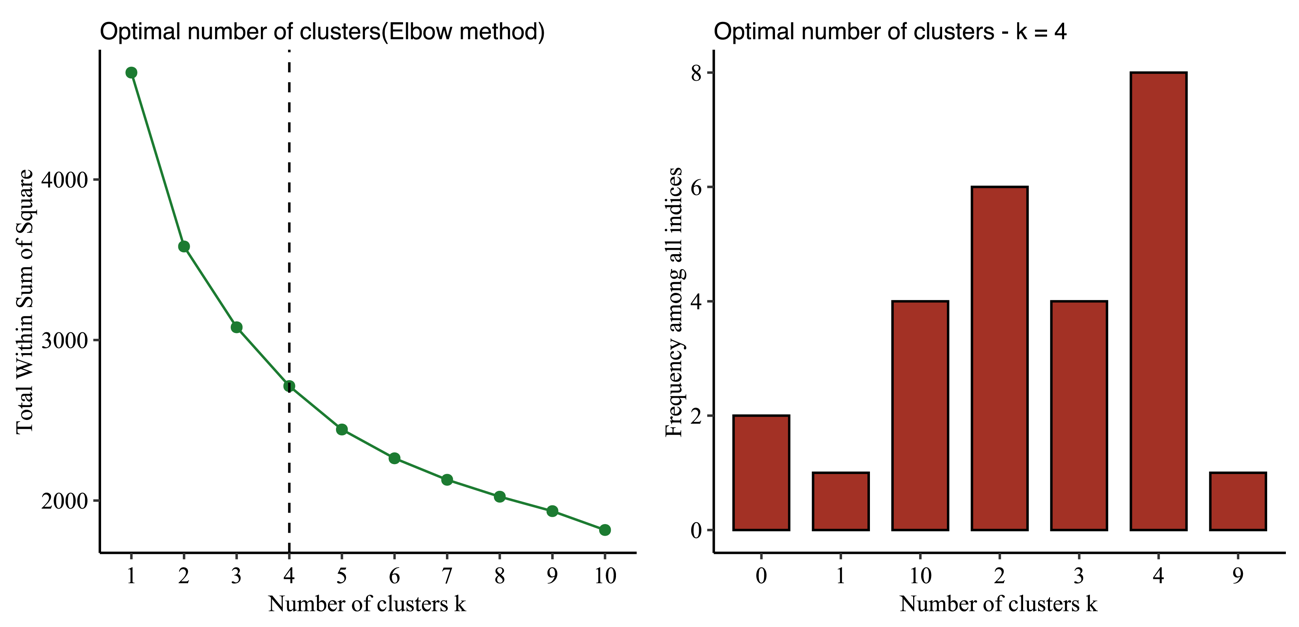


**Fig. S2**. The aggregation method of clusters is median and the optimal number of clusters is determined by the elbow method. (A) The slow down on the rate of the decreased total within-cluster sum of square indicated four being the optimal number of clusters. (B) the frequency numbers indicated four being the most reasonable cluster numbers.

# Supplementary Tables

Table S1. Analysis of variance in systolic blood pressure (SBP)

|  | Estimate | Standard Error | t value | Adj. p value |
| --- | --- | --- | --- | --- |
| Intercept | 103.77 | 3.26 | 31.84 | <0.001 |
| Age | 0.38 | 0.05 | 7.23 | <0.001 |
| MOD vs. MARD | 3.65 | 1.58 | 2.31 | 0.021 |
| SIDD vs. MARD | 5.92 | 1.59 | 3.73 | <0.001 |
| SIRD vs. MARD | 0.54 | 1.57 | 0.35 | 0.729 |

Table S2. Analysis of variance in diastolic blood pressure (DBP)

|  | Estimate | Standard Error | t value | Adj. p value |
| --- | --- | --- | --- | --- |
| Intercept | 85.82 | 1.93 | 44.52 | <0.001 |
| Age | -0.17 | 0.03 | -5.60 | <0.001 |
| MOD vs. MARD | 3.14 | 0.94 | 3.35 | <0.001 |
| SIDD vs. MARD | 5.82 | 0.94 | 6.21 | <0.001 |
| SIRD vs. MARD | 1.52 | 0.93 | 1.63 | 0.104 |

Table S3. Analysis of variance in estimated glomerular filtration rate (eGFR)

|  | Estimate | Standard Error | t value | Adj. p value |
| --- | --- | --- | --- | --- |
| Intercept | 146.59 | 3.30 | 44.41 | <0.001 |
| Age | -1.00 | 0.05 | -18.77 | <0.001 |
| MOD vs. MARD | -0.86 | 1.61 | -0.53 | 0.594 |
| SIDD vs. MARD | 5.38 | 1.61 | 3.35 | <0.001 |
| SIRD vs. MARD | -5.80 | 1.60 | -3.63 | <0.001 |

Table S4. Analysis of variance in uric acid (UA)

|  | Estimate | Standard Error | t value | Adj. p value |
| --- | --- | --- | --- | --- |
| Intercept | 326.48 | 15.68 | 20.83 | <0.001 |
| Age | 0.16 | 0.25 | 0.65 | 0.516 |
| MOD vs. MARD | 25.98 | 7.64 | 3.40 | <0.001 |
| SIDD vs. MARD | -26.00 | 7.64 | -3.40 | <0.001 |
| SIRD vs. MARD | 32.34 | 7.57 | 4.27 | <0.001 |

Table S5. Analysis of variance in urine albumin creatinine ratio (UACR)

|  | Estimate | Standard Error | t value | Adj. p value |
| --- | --- | --- | --- | --- |
| Intercept | -167.21 | 107.91 | -1.55 | 0.122 |
| Age | 4.53 | 1.74 | 2.61 | 0.009 |
| MOD vs. MARD | 104.71 | 52.44 | 2.00 | 0.046 |
| SIDD vs. MARD | 71.78 | 52.71 | 1.36 | 0.174 |
| SIRD vs. MARD | 50.73 | 52.49 | 0.967 | 0.334 |

Table S6. Analysis of variance in total cholesterol (TC)

|  | Estimate | Standard Error | t value | Adj. p value |
| --- | --- | --- | --- | --- |
| Intercept | 5.67 | 0.24 | 24.03 | <0.001 |
| Age | -0.02 | 0.01 | -4.05 | <0.001 |
| MOD vs. MARD | -0.13 | 0.12 | -1.13 | 0.261 |
| SIDD vs. MARD | 0.38 | 0.11 | 3.33 | <0.001 |
| SIRD vs. MARD | -0.526 | 0.11 | -4.60 | <0.001 |

Table S7. Analysis of variance in total glucose (TG)

|  | Estimate | Standard Error | t value | Adj. p value |
| --- | --- | --- | --- | --- |
| Intercept | 1.88 | 0.36 | 5.17 | <0.001 |
| Age | -0.01 | 0 | -1.04 | 0.296 |
| MOD vs. MARD | 0.790 | 0.18 | 4.45 | <0.001 |
| SIDD vs. MARD | 0.81 | 0.18 | 4.59 | <0.001 |
| SIRD vs. MARD | 0.13 | 0.18 | 0.73 | 0.465 |

Table S8. Analysis of variance in high-density lipoprotein (HDL)

|  | Estimate | Standard Error | t value | Adj. p value |
| --- | --- | --- | --- | --- |
| Intercept | 1.24 | 0.07 | 17.05 | <0.001 |
| Age | 0.00 | 0.00 | 1.19 | 0.233 |
| MOD vs. MARD | -0.24 | 0.04 | -6.74 | <0.001 |
| SIDD vs. MARD | -0.18 | 0.04 | -5.14 | <0.001 |
| SIRD vs. MARD | -0.17 | 0.04 | -4.78 | <0.001 |

Table S9. Analysis of variance in low-density lipoprotein (LDL)

|  | Estimate | Standard Error | t value | Adj. p value |
| --- | --- | --- | --- | --- |
| Intercept | 3.53 | 0.18 | 19.28 | <0.001 |
| Age | -0.01 | 0.00 | -3.97 | <0.001 |
| MOD vs. MARD | -0.08 | 0.09 | -0.89 | 0.373 |
| SIDD vs. MARD | 0.29 | 0.09 | 3.273 | <0.001 |
| SIRD vs. MARD | -0.33 | 0.09 | -3.70 | <0.001 |
